# Investigation of tumor hypoxia using a two-enzyme system for *in vitro *generation of oxygen deficiency

**DOI:** 10.1186/1748-717X-6-35

**Published:** 2011-04-10

**Authors:** Vasileios Askoxylakis, Gunda Millonig, Ute Wirkner, Christian Schwager, Shoaib Rana, Annette Altmann, Uwe Haberkorn, Jürgen Debus, Sebastian Mueller, Peter E Huber

**Affiliations:** 1 Department of Radiooncology and Radiation Therapy, University of Heidelberg, Heidelberg, Germany; 2 Center for Alcohol Research and Salem Medical Center, University of Heidelberg, Heidelberg, Germany; 3 Department of Radiation Therapy, German Cancer Research Center, Heidelberg, Germany; 4 Department of Nuclear Medicine, University of Heidelberg, Heidelberg, Germany; 5 Clinical Cooperation Unit Nuclear Medicine, German Cancer Research Center, Heidelberg, Germany

## Abstract

**Background:**

Oxygen deficiency in tumor tissue is associated with a malign phenotype, characterized by high invasiveness, increased metastatic potential and poor prognosis. Hypoxia chambers are the established standard model for *in vitro *studies on tumor hypoxia. An enzymatic hypoxia system (GOX/CAT) based on the use of glucose oxidase (GOX) and catalase (CAT) that allows induction of stable hypoxia for *in vitro *approaches more rapidly and with less operating expense has been introduced recently. Aim of this work is to compare the enzymatic system with the established technique of hypoxia chamber in respect of gene expression, glucose metabolism and radioresistance, prior to its application for *in vitro *investigation of oxygen deficiency.

**Methods:**

Human head and neck squamous cell carcinoma HNO97 cells were incubated under normoxic and hypoxic conditions using both hypoxia chamber and the enzymatic model. Gene expression was investigated using Agilent microarray chips and real time PCR analysis. ^14^C-fluoro-deoxy-glucose uptake experiments were performed in order to evaluate cellular metabolism. Cell proliferation after photon irradiation was investigated for evaluation of radioresistance under normoxia and hypoxia using both a hypoxia chamber and the enzymatic system.

**Results:**

The microarray analysis revealed a similar trend in the expression of known HIF-1 target genes between the two hypoxia systems for HNO97 cells. Quantitative RT-PCR demonstrated different kinetic patterns in the expression of carbonic anhydrase IX and lysyl oxidase, which might be due to the faster induction of hypoxia by the enzymatic system. ^14^C-fluoro-deoxy-glucose uptake assays showed a higher glucose metabolism under hypoxic conditions, especially for the enzymatic system. Proliferation experiments after photon irradiation revealed increased survival rates for the enzymatic model compared to hypoxia chamber and normoxia, indicating enhanced resistance to irradiation. While the GOX/CAT system allows independent investigation of hypoxia and oxidative stress, care must be taken to prevent acidification during longer incubation.

**Conclusion:**

The results of our study indicate that the enzymatic model can find application for *in vitro *investigation of tumor hypoxia, despite limitations that need to be considered in the experimental design.

## Background

Reduced oxygen levels are measured in several solid tumors mainly as result of tumor outgrowing the existing vasculature but also as result of structural and functional disturbances of tumor vasculature [1]. In particular, tumor blood vessels that are newly formed during angiogenesis are highly irregular and possess incomplete endothelial linings and basement membranes, as well as arteriovenous shunts, resulting in disturbances of blood flow and oxygen delivery [2]. Tumor hypoxia is associated with a more aggressive neoplastic phenotype, characterized by high invasiveness and increased metastatic potential. Genes with key-role in metastatic processes, such as lysyl oxidase (LOX), met proto-oncogene (MET) and c-X-c chemokine receptor 4 (CXCR4) have been identified to be upregulated under hypoxic conditions [3,4]. In regard to therapy outcome and prognosis, hypoxic regions within a solid tumor are characterized by increased resistance towards chemotherapy or radiotherapy. In particular, oxygen deficiency upregulates the expression of the multidrug resistance gene (MDR1), leading to efflux of chemotherapeutic drugs [5]. In respect to radiation therapy both chemical and biological mechanisms are found to be important for increased radioresistance. Oxygen deficiency disturbs the radiolysis of H_2_O leading to reduced production of reactive species that are cytotoxic [6]. Furthermore, hypoxia promotes the activation of the hypoxia inducible factor-1 (HIF-1), a heterodimeric transcription factor that upregulates the expression of genes involved in angiogenesis and tumorigenesis [7].

The fact that tumor hypoxia is associated with increased therapy resistance and poor prognosis reveals the necessity for extensive and detailed investigation of biological mechanisms associated with oxygen deficiency. The established method for *in vitro *investigation of tumor hypoxia is the exposure of cultured cells to defined, oxygen deficient gaseous environments. The most common apparatus used for this purpose is the hypoxia chamber. However this approach has critical limitations, mainly in regard to oxygen diffusion and equilibration. In particular, within a hypoxia chamber oxygen reaches the cell surface after a protracted process, including transport in the chamber, passing through the material of the cell culture plate, solubility depended entering the culture medium at the gas-medium interface and diffusion through the medium to the cell surface. Oxygen transport kinetic studies in the past have revealed required time periods of about 30 min for equilibration of pO_2 _between the gas inside and outside of the culture plate and more than 3 h for equilibration of the pO_2 _between the medium inside the plate and the gas outside of it [8].

Recently an alternative way to generate *in vitro *oxygen-deficient conditions has been evaluated [9,10]. This system is based on the use of the enzymes glucose oxidase (GOX) and catalase (CAT). Addition of glucose oxidase into the cell culture medium removes oxygen by oxidizing glucose. The reaction leads to generation of hydrogen peroxide, which is then removed by catalase, in order to prevent cytotoxic effects due to accumulation. This enzymatic system was found to induce rapid depletion of oxygen within minutes at a defined rate. Oxygen concentration in the cultured medium is reported to be dependent by two factors: the activity of glucose oxidase and the medium volume. GOX activity has an influence on the depletion rate of oxygen, while medium volume affects the diffusion distance of oxygen from gas-medium interface to the cells. Experiments have revealed that at defined GOX activity and medium volume, controlled oxygen depletion can be achieved and also stably maintained for at least 12-24 h [10].

Aim of the present work is to investigate the effects of rapidly induced hypoxia on cellular processes using the enzymatic GOX/CAT system in comparison to the established method of hypoxia chamber. Since hypoxia is known to be a feature of human head and neck squamous cell carcinoma [11], the HNSCC cell line HNO97 was chosen for investigation under normoxic and hypoxic conditions using a hypoxia chamber and the enzymatic model. We focused on three aspects: gene expression, glucose metabolism and radioresistance. Gene expression was investigated using Agilent microarray chip analysis and real time PCR. Cellular glucose metabolism was assessed with ^14^C-FDG uptake assays and proliferation experiments after photon irradiation were carried out for investigation of hypoxia induced radioresistance. The results of our study indicate that the enzymatic GOX/CAT system is an attractive alternative technique for *in vitro *investigation of tumor hypoxia.

## Methods

### Cell culture

The human head and neck squamous cell carcinoma cell line HNO97 [12] was cultivated in Dulbecco's Modified Eagle's Medium (DMEM containing 4.5 g/L glucose and 58 ng/L L-glutamine but no sodium pyruvate) supplemented with 10% (v/v) fetal calf serum (Gibco, Invitrogen Life Technologies) at 37°C in a 5% CO_2 _incubator.

### *In vitro *enzymatic and non-enzymatic hypoxia induction

#### Enzymatic hypoxia

Hypoxia medium was prepared by diluting glucose oxidase and catalase at a constant 1:10 ratio in cell culture medium (both Sigma cat. No. C3155 and G0543). Enzyme activities of stock solutions were 3 mM/s for GOX and 998 s^-1 ^for CAT. To obtain a defined, stable oxygen concentration of 2% on cell surface stock solutions were diluted by 1:10,000 for GOX and 1:1,000 for CAT. The medium volumes used were 2.5 ml for 6-well plates and 10.63 ml for 10 cm cell culture plates and the cells were incubated at 37°C. Previous experiments using a computer-driven oxygen electrode Oxi 325-B (WTW, Weilheim, Germany) for oxygen measurement have revealed that at those conditions 2% hypoxia was rapidly induced within 15 min and maintained over 24 h [10]. For incubation periods longer than 24 h medium was replaced by pre-equilibrated hypoxic medium to maintain nutrients and substrates such as glucose.

### Hypoxia chamber

Cells cultivated in 6-well plates or 10 cm cell culture dishes under a layer of exactly 2.5 ml and 10.63 ml cell culture medium respectively were placed in a hypoxia chamber. The chamber was flushed with 2% O_2_/5% CO_2_/93% N_2 _gas mixture for 5 min, sealed and kept at 37°C. For longer incubation periods the chamber was refilled after 24 h to ensure constant oxygen concentrations.

### Real time quantitative PCR

Total cellular RNA was isolated from confluent head and neck squamous cell carcinoma HNO97 cells using Trizol (TRIzol Reagent, Invitrogen #15596-018) according to manufacturer instructions. RNA concentration was measured with a NanoDrop spectrophotometer (ND-1000 PeqLab Biotechnologie GmbH, Germany). 500 ng was transcribed into DNA using M-MLV reverse transcriptase, 50 pmol random hexamer and 100 pmol of oligo(dT) primers (Promega, Madison, WI, USA). Quantification of relative mRNA transcript levels of human carbonic anhydrase IX (CA9) and lysyl oxidase (LOX) was performed on a StepOnePlus™ Real-Time PCR System (Applied Biosystems), applying the TaqMan methodology. Normalization was performed using B2 microglobulin (B2M) as endogenous control. Primers were obtained from Applied Biosystems (Foster City, CA, USA).

### Gene expression

Gene expression of HNO97 cells under normoxic and hypoxic conditions was investigated using whole human genome microarrays. Total RNA from time points t = 0, and after 24 h incubation under hypoxic conditions (2% O_2_) using the hypoxia chamber and the GOX/CAT system was investigated. To determine the influence of cell density on gene expression, microarray analysis was also performed for RNA isolated from cells incubated for the same time period (24 h) under normoxic conditions. For bioinformatical-analysis a step-wise approach was applied: Weak signals, below the intensity of spike-in linearity, were excluded, quantile normalization was performed on background-subtracted signal intensities, ratios were calculated by arithmetic mean normalization of control group (t = 0 or normoxia t = 24 h) versus all samples. Afterwards Log2 of ratios was calculated.

### Microarray processing and data extraction

Genome-wide expression profiling was carried out using whole human genome 4 × 44 k oligo microarrays (Agilent, G4112F). Linear amplification from 500 ng total RNA and spike-in-controls (Agilent #5188-5282) was performed using the Agilent "Low RNA Input Linear Amplification Kit Plus, one colour" (#5188-5339). During this process the amplified RNA was directly labelled by incorporation of Cy3-labelled CTP. Labelled RNA was purified with "RNeasy" mini spin columns (Qiagen #74104) and 1.65 μg labelled RNA was used for chemical fragmentation and hybridisation (Gene expression hybridization kit, Agilent #5188-5242). Assembly of the gasket/slide-sandwich in the hybridisation chamber (Agilent, #G2534A) was performed according to manufacturer instructions. For hybridisation, slide-sandwiches were rotated at 10 rpm and 65°C for 16 h. Slides were washed 1 min in GE Wash Buffer 1 at RT, 1 min in GE Wash Buffer 2 at RT (Agilent, #5188-5325, 5188-5326) and 30 sec in Acetonitril at RT on a magnetic stirrer. Slides were scanned in an Agilent Microarray Scanner. Data extraction of the resulting array images was performed using the "Feature Extraction" software (Agilent, Version 9.1) and SUMO (Christian Schwager, http://angiogenesis.dkfz.de/oncoexpress/software/sumo/) was used for statistical analysis, two-class t-tests and GO-analysis. Pathway analysis was performed based on information available on cellular signalling processes from a curated database on signalling networks and systems biology package (Metacore, Genego, St Joseph, MI, USA, http://www.genego.com).

### FDG uptake

After trypsinisation 5 × 10^4 ^HNO97 cells were seeded in 6-well plates. Cells were incubated in DMEM + 10% FCS for 24 h. Medium was removed and the cells were incubated for 6 h and 24 h under normoxic and hypoxic conditions (2% O_2_), using the enzymatic GOX/CAT system and a hypoxia chamber. Subsequently, FDG uptake experiments were performed in glucose-free DMEM medium as described in the literature [13]. In particular, after 30 min of pre-incubation in glucose-free medium, 37 kBq 2-fluoro-2-deoxy-D-[U-14C] glucose (FDG; Amersham-Buchler; specific activity 10.8 GBq/mmol; radioactive concentration 7.4 MBq/ml; radiochemical purity 99.3%) per ml medium and cold FDG were added to a final concentration of 0.1 mM. Cells were incubated for 10 min with radioactive FDG and thereafter the medium was removed and the cells were washed three times with ice-cold PBS. Cells were then lysed on ice with 1M NaOH. The lysates were counted on a scintillation counter. The viable cell number was determined by a Vi-Cell™ XR Cell Viability Analyzer (Beckman Coulter). Radioactive FDG uptake was calculated as % applied dose per 10^6 ^cells. The experiment was performed in triplicate and repeated twice.

### Cell viability

50,000 human head and neck squamous cell carcinoma HNO97 cells were seeded in 6-well plates and incubated overnight at standard conditions and subsequently for 24 h under normoxia and hypoxia (2% O_2_) using both the enzymatic GOX/CAT system and a hypoxia chamber. Thereafter, cells were trypsinized and their viability was investigated with automated trypan blue viability assays using a Vi-Cell™ XR Cell Viability Analyzer (Beckman Coulter).

### Cell irradiation and proliferation assay

Proliferation assays were performed as described in the literature [14]. 50,000 human head and neck squamous cell carcinoma HNO97 cells were seeded in 6-well plates and incubated overnight at standard conditions. The cells were then incubated for 24 h at normoxic and hypoxic conditions using both the enzymatic GOX/CAT system and a hypoxia chamber. Irradiation with 6 MV X-rays (Mevatron Siemens) at a dose of 4 Gy was performed and further incubation for 72 h at the same conditions as before irradiation was carried out. Thereafter the cells were trypsinized and counted with a Vi-Cell™ XR Cell Viability Analyzer (Beckman Coulter). Non-irradiated cells were incubated at the same conditions. The ratio vital irradiated/non-irradiated cells, which represents the proportion of vital cells after irradiation, compared to the non-irradiated control was calculated.

### Statistical analysis

Statistical analysis of the genomics was performed with SUMO (Christian Schwager, http://angiogenesis.dkfz.de/oncoexpress/software/sumo/) using two-class t-tests. Data of FDG-uptake and cell proliferation assays were analyzed employing the Student t-test. Significance was assumed at p < 0.05.

## Results

### Expression profiling

**Figure 1:**
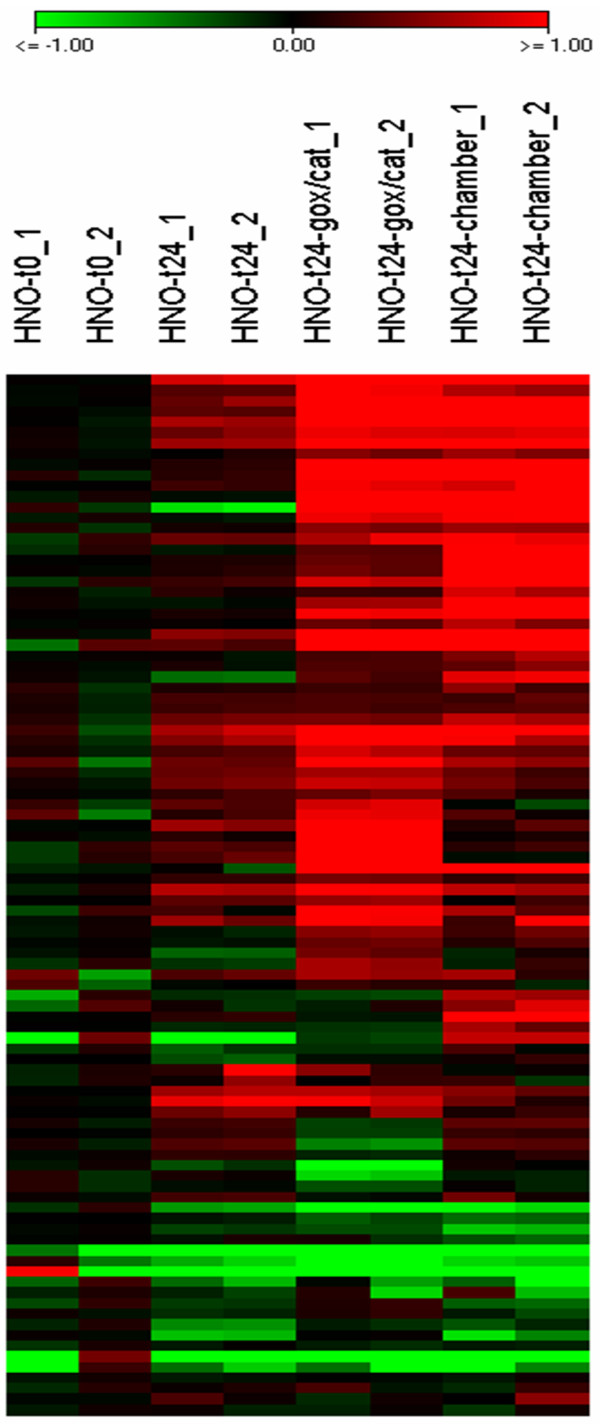
**Transcriptomics from HNO97 head and neck squamous cell carcinoma cells under normoxia and hypoxia**. Gene expression pattern of known and validated HIF-1 target genes [15] before and after 24 h incubation under normoxic and hypoxic conditions (2% O_2_) using the enzymatic GOX/CAT system and a hypoxia chamber. The colour scale encodes differential regulation of genes from green (≤- 2-fold downregulated vs. reference normoxia t = 0 RNA) to red (≥+ 2-fold upregulated vs. reference normoxia t = 0 RNA).

After 24 h incubation in a hypoxia chamber and with the GOX/CAT system the expression of known and validated HIF-1 target genes as described in the literature [15] was evaluated for HNO97 cells. The experiments demonstrated a similar trend in the expression of known HIF-1 target genes for both systems (Figure 1). An overview of the expression of known HIF-1 target genes for HNO97 cells is presented in Additional file 1.

**Figure 2:**
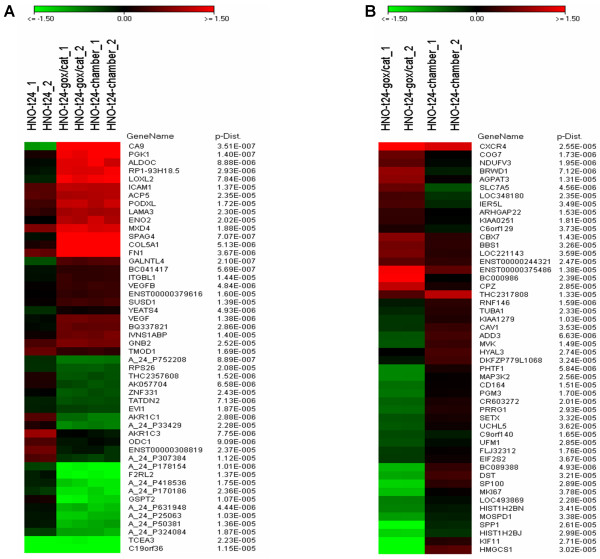
**Strongest and differentially regulated genes**. (A) Strongest regulated genes in HNO97 cells under hypoxia and respective p-values. (B) Differentially regulated genes between the GOX/CAT system and hypoxia chamber in HNO97 cells and respective p-values. The colour scale encodes differential regulation of genes from green (downregulated vs. reference normoxia t = 24 RNA) to red (upregulated vs. reference normoxia t = 24 RNA).

In order to identify the strongest regulated genes for HNO97 cells under hypoxia, a 2-class t-test was performed. The 50 strongest regulated genes and the respective p-values for the GOX/CAT system and the hypoxia chamber compared to normoxic cells at 24 h are presented in Figure 2A. Among them 7 genes are known HIF-1 target genes (CA9, PGK1, ALDOC, COL5A1, FN1, VEGF, ENO2), while 4 further genes are described to be associated with hypoxia (AKR1C3, ICAM1, LOXL2, LAMA3).

Differentially regulated genes between the two hypoxic systems in HNO97 cells were identified performing a two-class t-test after normalization against the control chips from the normoxic cells at t = 24 h. The statistical analysis revealed the 50 strongest differentially regulated genes (Figure 2B). Among them only 1 gene was found to be a HIF-1 target (CXCR4).

**Figure 3:**
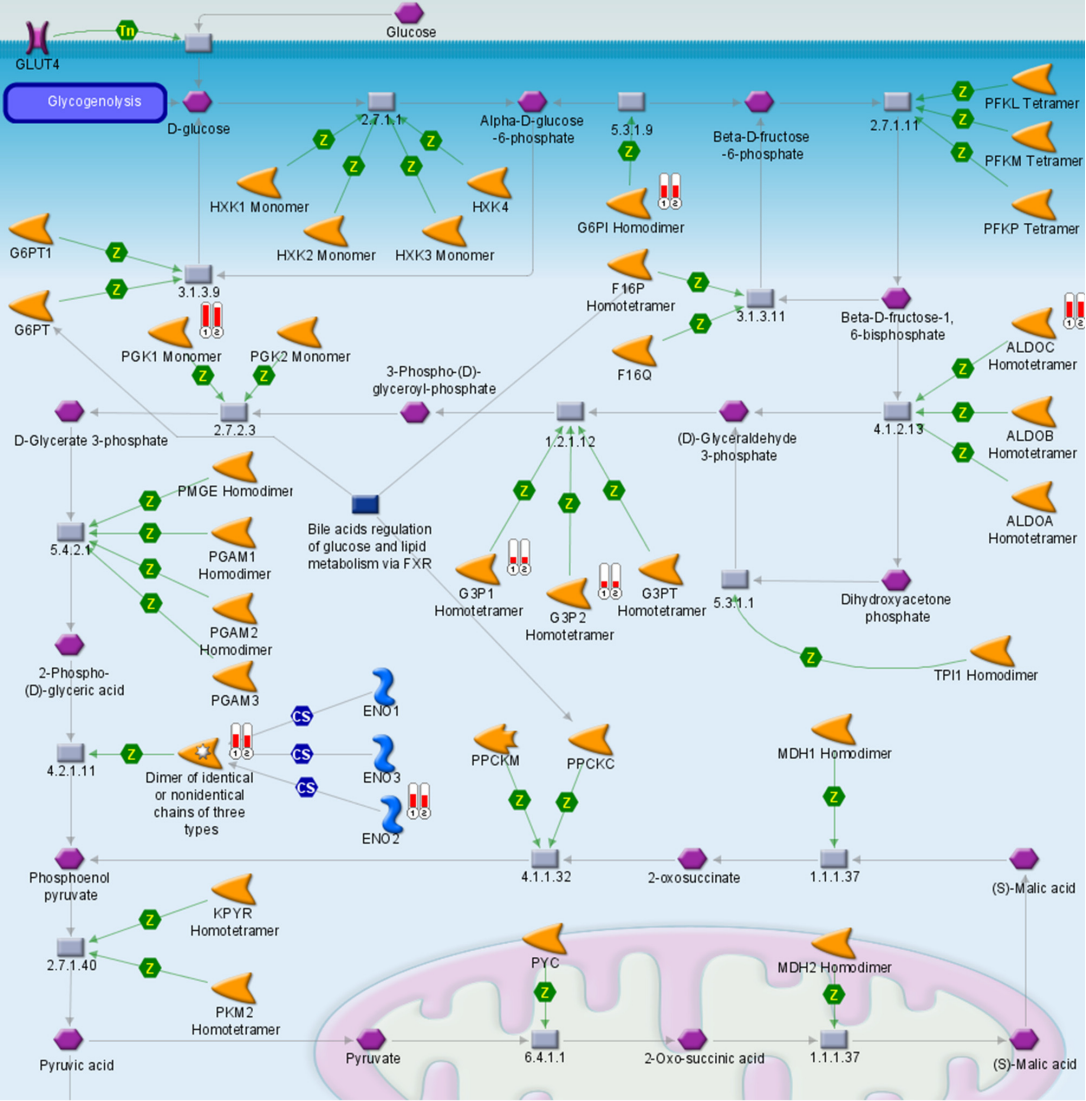
**Pathway analysis of hypoxia regulated genes using a hypoxia chamber and the enzymatic GOX/CAT system**. The genes PGK1, PGM1, SDS, ENO2, ALDOC, GAPDH, GPI and HKDC1 were upregulated for both hypoxia systems (Thermometer 1: Hypoxia chamber, Thermometer 2: GOX/CAT system). Those genes are involved in glycolytic pathways. p = 4 × E-5.

Functional groups of hypoxia regulated genes for both systems were identified by assigning them to biological function GO-terms. The most probably regulated GO-Term was Glycolysis (p = 4 × E-5), which is shown in Figure 3.

### RT-PCR

**Figure 4:**
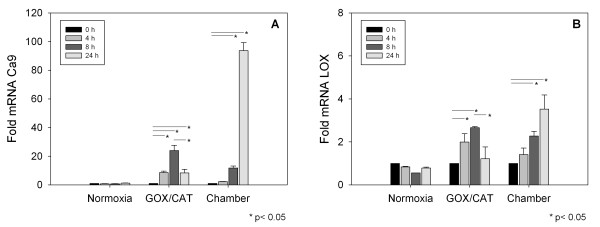
**Quantitative RT-PCR analysis of the expression of hypoxia regulated genes**. Expression of carbonic anhydrase IX (CA9) (A) and lysyl oxidase (LOX) (B) in head and neck squamous cell carcinoma cells HNO97 under normoxia and hypoxia (2% O_2_) using the enzymatic GOX/CAT system and a hypoxia chamber. mRNA levels were measured by quantitative real time PCR. Columns, average from three independent measurements and show relative expression levels compared with cells at time point t = 0; Bars, SD. * p < 0.05.

In addition to microarray analysis (Additional file 1), quantification of the tumor hypoxia regulated genes CA9 and LOX was performed with real time PCR. To evaluate the gene expression under hypoxic conditions over time, HNO97 cells were incubated for time periods of 4 h, 8 h and 24 h under normoxic conditions, in the hypoxia chamber and with the GOX/CAT system. The RT-PCR experiments demonstrated an upregulation of the tumor hypoxia dependent genes for both systems (p < 0.05). However, time kinetic of the gene expression was different between the slow hypoxia chamber and the rapid hypoxia GOX/CAT system. In particular, the highest level of CA9 and LOX expression was shown at 8 h incubation for the enzymatic system and then decreased, while a continuous increase over time for the incubation period was identified for the hypoxia chamber (Figure 4).

### FDG uptake

**Figure 5:**
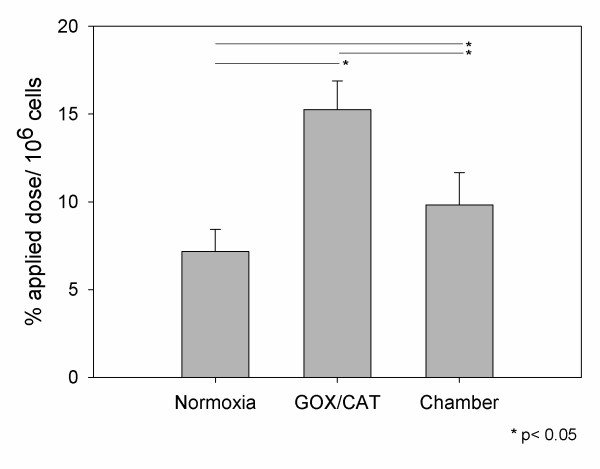
**Glucose metabolism**. Uptake of FDG in HNO97 cells incubated for 24 h under normoxic and hypoxic conditions (2% O_2_) using the GOX/CAT system and a hypoxia chamber. Mean values and standard deviation. * p < 0.05.

Fluorodeoxyglucose (FDG) uptake experiments were carried out in order to evaluate the influence of hypoxia in the metabolic activity of HNO97 cells. For these experiments cells were cultivated under normoxia or hypoxia (2% O_2_) for 6 h and 24 h and subsequently radioactive FGD was shortly applied on the cells and the uptake was determined. These studies demonstrated an enhanced FDG uptake under hypoxia. After 6 h cultivation the FDG uptake was significantly increased for the GOX/CAT system (p < 0.05). In regard to the hypoxia chamber, only a slight increase was noticed compared to normoxia (Table 1). After 24 h cultivation a significant FDG uptake enhancement was noticed for both hypoxic systems compared to normoxia (p < 0.05). Still, the enhancement of FDG uptake was higher for the rapid hypoxia inducing enzymatic model (p < 0.05) compared to the slower hypoxia inducing chamber (Figure 5). The ratios of FDG uptake under hypoxia to FDG uptake under normoxia are presented for both hypoxia systems in Table 1.

**Table 1:** Glucose metabolism

Ratio FDG-uptake hypoxia/FDG-uptake normoxia	GOX/CAT	Chamber
6 h	1.87	1.09
24 h	2.13	1.37

Uptake of FDG in HNO97 cells after cultivation for 6 h and 24 h under normoxic and hypoxic conditions (2% O_2_) using the GOX/CAT system and a hypoxia chamber. Values of the ratio FDG-uptake under hypoxia to FDG-uptake under normoxia.

### Cell viability

Cell viability was investigated with a Vi-Cell™ XR Cell Viability Analyzer (Beckman Coulter) to determine whether hypoxia at the applied conditions might cause cell death. Cell number evaluation after 24 h cultivation using GOX/CAT and a hypoxia chamber showed absolute cell numbers of about 70% and 90% of the absolute cell number after 24 h cultivation under normoxia. Trypan blue analysis revealed that hypoxia did not induce cell death at the applied conditions. In particular, no significant difference was noticed in the percentage of unvital cells (5-10%) for both normoxia and hypoxia using GOX/CAT or chamber. Furthermore, microscopy studies showed that the cells were still attached and morphologically intact under the hypoxic conditions used (data not shown).

### Cell proliferation after photon irradiation

**Figure 6:**
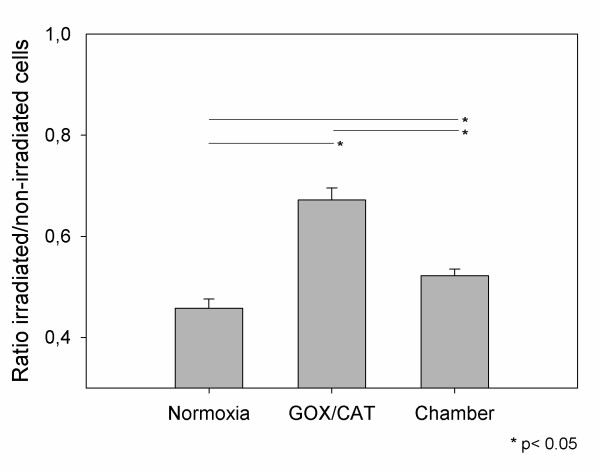
**In vitro cell response to photon irradiation in the 72-h proliferation assay**. Cells were incubated for 24 h under normoxia and hypoxia (2% O_2_) using the GOX/CAT system and a hypoxia chamber. The ratio vital treated to vital untreated cells was determined. Mean values and standard deviation. * p < 0.05.

Proliferation of HNO97 cells was investigated for normoxia and hypoxia after photon irradiation at a single dose of 4 Gy. Vital cell number was measured and the ratio vital irradiated/non-irradiated cells, was determined. This ratio represents the proportion of vital cells after irradiation compared to the non-irradiated control. The proliferation assays revealed higher ratios when HNO97 cells were incubated under hypoxic conditions (p < 0.05), indicating an enhanced cell resistance to the applied radiation dose. This ratio was only slightly enhanced for the slow-onset hypoxia chamber system but was higher for the enzymatic GOX/CAT system (p < 0.05) (Figure 6).

To determine whether different cell confluences, as result of different cell growth rates under normoxia and hypoxia, had an influence on irradiation outcome, proliferation experiments after photon irradiation with 4 Gy were performed for various cell confluences under normoxia. These experiments revealed no significant differences in irradiation outcome within the cell number range that was measured for normoxia, hypoxia chamber and GOX/CAT (50,000 to 200,000 cells) at the time of irradiation (Additional file 2).

## Discussion

The microenviroment within a solid tumor has an extensive influence on the outcome of cancer treatment and the prognosis of the disease. Tumor hypoxia affects the behaviour of tumor cells and is associated with poor prognosis and reduced overall survival [16]. This fact reveals the need for a detailed study of biological effects under reduced oxygen levels. The most common technique used to investigate *in vitro *tumor hypoxia is the hypoxia chamber. However, this approach has limitations. The method requires special technical equipment while it has been shown that it leads to a slow onset of hypoxia that might influence the correlation between changes in oxygen concentration and kinetic of hypoxia dependent biological events.

An alternative to hypoxia chamber represents the enzymatic GOX/CAT system, which has been shown to rapidly induce *in vitro *hypoxia. The GOX/CAT system has been employed in the past in various studies. In particular, Baumann et al. have applied the enzymatic system for investigation of the effects of the hypoxia-targeted prodrug KS119 [9,17]. Furthermore, Zitta et al. used GOX/CAT for rapidly induction of hypoxia and investigated the influence of mild hypothermia and postconditioning with catalase on hypoxia-mediated cell damage [18], as well as the potential cytoprotective properties of different sevoflurane conditioning strategies on a human neuronal cell culture model [19]. In addition, Owegi et al. applied the GOX/CAT technique to test macrophage activity under various O_2 _and H_2_O_2 _concentrations, as presented under infection conditions [20]. All these studies have demonstrated a rapid decrease of oxygen concentration using glucose oxidase and catalase but provided only limited comparisons to the established hypoxia chamber technique. Therefore, in the present study we evaluated the enzymatic GOX/CAT system in direct comparison to the established hypoxia chamber technique for investigation of different biological events, including gene expression, glucose uptake and radioresistance at a defined O_2 _concentration.

The conditions for *in vitro *generation of hypoxia at a level of 2% were carefully chosen in concert with the results of previous studies. In particular, evaluation of oxygen concentration using a computer-driven oxygen electrode revealed that at the conditions used for our experiments 2% hypoxia was rapidly induced within 15 min and maintained over 24 h [10]. Since oxygen transport studies using hypoxia chambers have revealed time periods of more than 3 h for equilibration of pO_2 _between the medium inside the plate and the gas outside of it, which even accelerated in the presence of cells [8], evaluation of both systems was performed after 24 h cell cultivation under hypoxic conditions to ensure that the observed biological events are not a result of differences in the oxygenation level. We further chose for our investigation a head and neck squamous cell carcinoma (HNSCC) cell line because there is strong evidence that hypoxia is an important microenvironment factor, which influences the response of HNSCC to therapy [21] and because the role of low oxygen tension has been extensively investigated for this cancer entity both in preclinical and in clinical studies [22,23].

Our experiments demonstrated comparable trends for both systems in regard to gene expression, glucose uptake and resistance towards radiation therapy. In particular, investigation of hypoxia related genes using microarray chip analysis in our study revealed a similar regulation trend for most known HIF-1 target genes for both the rapid enzymatic GOX/CAT system and the hypoxia chamber after 24 h of hypoxia (Figure 1). The expression of prominent hypoxia dependent genes, such as carbonic anhydrase IX (CA9) and lysyl oxidase (LOX) was additionally to microarray analysis quantified by real time PCR. These genes were chosen for analysis not only because it is known that they are hypoxia regulated, but also because various studies have reported prognostic values for them in head and neck squamous cell carcinoma [24,25]. Microarray analysis in our study indicated CA9 and LOX activation both in the chamber and the enzymatic system after 24 h, while CA9 showed stronger activation than LOX. Quantification through real time PCR demonstrated different kinetic patterns between the two hypoxia systems (Figure 4). Particularly, although both genes were upregulated under hypoxic conditions the upregulation peak was reached earlier for the rapid enzymatic GOX/CAT system and decreased thereafter, compared to the hypoxia chamber that showed a continuous increase of gene expression over 24 h. Our results are in concert with the results of previous studies using the GOX/CAT system [10]. Millonig et al. have shown that a fast onset of hypoxia using the enzymatic system leads to rapid induction of HIF-1 that later disappears although the cells remain under stable hypoxia. In contrast, cell exposure to the same oxygen concentration using a conventional hypoxia chamber causes a late onset and continuous upregulation of HIF-1 over a time period of 24 h. These results led the authors to the conclusion that HIF-1 responds rather to oxygen decrements than to absolute hypoxia, a hypothesis that might also explain the different kinetic patterns of the HIF-1-target genes CA9 and LOX as demonstrated in our study.

In regard to glucose metabolism, uptake experiments of fluorodeoxyglucose (FDG) revealed an enhanced cellular uptake for both the enzymatic and the chamber system (Figure 5), which increased with time progression (Table 1). This result is expected, since it is known that hypoxia is associated with a reprogrammed cellular metabolism, characterized by enhanced uptake of glucose for use as anabolic and catabolic substrate. The enhanced FDG uptake is supported by a HIF-1 dependent activation of the transcription of SLC2A1 and SLC2A3 genes, which encode the glucose transporters GLUT1 and GLUT3 respectively. Furthermore, HIF-1 activates the transcription of the HK1 and HK2 genes, which encode for hexokinase, an enzyme that phosphorylates FDG and represents the first enzyme of the Embden-Meyerhoff (glycolytic) pathway [26,27]. The role of HIF-1 in further metabolisation of glucose has been extensively investigated in previous studies. In particular, it has been shown that glycolytic enzymes which metabolize glucose to pyruvate, and lactate dehydrogenase A (LDHA) which further converts pyruvate to lactate are regulated by HIF-1, promoting ATP production through increased anaerobic glycolysis under hypoxic conditions [28]. The results of our study demonstrate that the new enzymatic GOX/CAT system affects glucose metabolism in a similar trend like the established hypoxia chamber. FDG uptake was increased for both systems, result that is in concert with the microarray analysis, which shows an upregulation of genes involved in glucose metabolism, such as SLC2A1, SLC2A3, HK1, HK2 and LDHA. The slower increase of FDG uptake for the hypoxia chamber, compared to GOX/CAT (Table 1) might be explained by different kinetics in the expression of HIF-1 target genes that are involved in glucose metabolism, considering the fact that further HIF-1 target genes, such as CA9 and LOX showed different expression kinetics for the two systems.

In regard to glucose metabolism, assignment of gene expression results to biological function gene ontology terms (GO-terms), demonstrated glycolysis to be the most probably regulated GO-term for both systems (Figure 3). This is expected since glycolysis is known to be the preferred route for energy production under conditions of oxygen deficiency. Although our results provide strong indications of glycolytic metabolism, further investigation of the ratio between lactate production and glucose consumption is needed in order to assess the balance between glycolytic and oxidative metabolism under normoxia and hypoxia using the GOX/CAT system. This is important, considering the fact that cancer cells are known to use glycolysis even under normoxic conditions. Since glycolysis can produce ATP at higher rates than oxidative phosphorylation [29] and tumor cells require fast energy production in order to support cell growth and survival, metabolic alterations in favour of glycolysis is noticed even under normoxia [30], demonstrating the complexity of pathways and mechanisms in respect to microenvironment adaptation of tumor cells.

The enzymatic GOX/CAT system has however a critical limitation that needs to be considered in experiments investigating glucose metabolism. Glucose oxidase (GOX) does not only consume oxygen but also leads to depletion of glucose in the incubation medium. Previous studies investigating *in vitro *FDG uptake in various cell lines have revealed that hypoglycemic conditions lead to an increased FDG uptake [31,32]. Furthermore, it has been shown that the enhanced transport activity caused by hypoglycemia is attributed to an increased expression of GLUT1 in the cell membrane [33]. Therefore, the GOX mediated glucose depletion might bias the results of metabolic experiments. The substrate consumption at various settings of the GOX/CAT system has been extensively evaluated [34]. Under our conditions, hypoxia could be stably maintained for about 24 h without replacing the medium and reagents, leading to a glucose decrease of about 10% [10]. For comparison, 5% equals the 24-hour glucose consumption of about 90 million exponentially growing tumor cells [35].

Subphysiologic levels of oxygen in the tumor lead to an up to 3-fold increase of resistance against antineoplastic strategies, such as radiation therapy [36]. The enhanced radioresistance is explained through a reduced production of cytotoxic reactive species and promotion of the upregulation of genes that protect the cells from irradiation [37]. Within our study we performed proliferation experiments after irradiation of the cells in order to investigate whether the enzymatic GOX/CAT system could be used for *in vitro *investigation of hypoxia related radioresistance. The comparison with the established hypoxia chamber revealed that at O_2 _concentration of 2% only a slight resistance increase was noticed for the hypoxia chamber system, while the GOX/CAT system showed a higher resistance to photon irradiation (Figure 6). The enhanced radioresistance for the rapid hypoxic strategy could be explained by an increased growth arrest in the G0/G1 phase of the cell cycle. It has been shown in the past that one of the genes that promote growth arrest in the G0/G1 phase via upregulation of p21 is heme oxygenase 1 (HMOX1) [38]. Our gene expression analysis revealed a strongly increased expression of HMOX1 for the GOX/CAT system compared to the hypoxia chamber (Log2 of 3.5 and 0.2, respectively), result that offers a possible explanation for the enhanced cytoprotection that needs to be further investigated.

The hypothesis of growth arrest through rapid hypoxia is supported by the results of viability experiments irrespective of irradiation. These experiments showed lower cell numbers for the hypoxic systems compared to normoxia. Trypan blue and microscopy analysis revealed however that the reduced cell number was not attributed to cell death. Our results might be explained by a reduced cell division and DNA synthesis, which has been described in previous studies using the enzymatic model [10].

The GOX/CAT system has some limitations. Besides the fact that GOX causes glucose depletion and therefore the results might be affected by substrate deprivation, the activity of GOX also leads to the production of D-gluconolactone, which may cause culture medium acidification. pH measurement during our studies with HNO97 cells revealed no significant acidification of the DMEM medium for the investigated time period of 24 h. However, using the GOX/CAT system for hypoxia induction on human umbilical vein endothelial cells (HUVEC) a rapid pH decrease to a level of about 4.0-4.5 was noticed, leading to RNA degradation and cell death (data not shown). The extracellular pH of malignant tumors is known to be acidic, within a range of 6.5 to 7.0, as a consequence of increased glucose metabolism and poor perfusion [39], promoting tumor cell invasion via several matrix remodeling systems, including metalloproteinases, lysosomal proteases and hyaluronidase [40,41]. However, the strong acidosis measured on HUVEC cells using the GOX/CAT system, can not only be attributed to physiologically induced acidocis. A possible explanation is a low buffer capacity of the HUVEC cell culture medium, which needs to be considered in the design of experiments using the GOX/CAT system. In order to minimize substrate depletion and gluconolactone production two strategies can be applied for incubation periods longer than 24 h. The first strategy is the replacement of the incubation medium by fresh, preequilibrated medium and the second is the use of larger volumes of medium, which will in turn increase the time to reach stable hypoxia [34].

Finally, it should be mentioned that the GOX/CAT system allows the additional generation and control of hydrogen peroxide independently of the degree of hypoxia [34,42]. Since reactive oxygen species play an important role during tumor growth and radiation therapy of tumors, this option may be highly interesting when studying the role of transcription factors such as HIF-1 that are both responsive to hypoxia but also reactive oxygen species.

## Conclusions

In conclusion, the results of our study indicate that the GOX/CAT system might be a useful tool for the *in vitro *investigation of tumor hypoxia. In comparison to the established hypoxia chamber techniques, the GOX/CAT approach can induce hypoxia rapidly and in a controlled manner, while it is inexpensive and does not require technical equipment. Despite limitations which should be considered in the experimental design, the enzymatic system represents an attractive and valuable alternative for studying biological events associated with tumor hypoxia that needs to be further investigated.

## Competing interests

The authors declare that they have no competing interests.

## Authors' contributions

VA and GM made substantial contributions to conception and design of the study, drafted the manuscript and gave approval of the final version. VA, GM, UW, CS and SR were involved in data analysis and data interpretation. AA, UH, JD, SM and PEH were involved in critically revising the manuscript for important intellectual content and gave approval of the final version. All authors have read and approved the final manuscript.

## Supplementary Material

Additional file 1 **Gene expression of known and validated HIF-1 target genes**. Gene expression in HNO97 cells under normoxic and hypoxic (2% O_2_) conditions using both a hypoxia chamber and the enzymatic GOX/CAT system. Mean values and standard deviation vs. reference normoxia t = 0 RNA. Click here for file

Additional file 2 **Cell response to photon irradiation for various cell numbers**. Cells were seeded in different confluences and incubated for 24 h under normoxia. Cell number was determined prior to irradiation. The ratio vital treated to vital untreated cells was determined 72 h after photon irradiation. Mean values and standard deviation. Click here for file
