# Supplementary figures and images for: Investigation of tumor hypoxia using a two-enzyme system for in vitro generation of oxygen deficiency
Source: Radiat Oncol. 2011 Apr 10;6:35. doi: 10.1186/1748-717X-6-35 (PMC3080288; doi:10.1186/1748-717X-6-35)

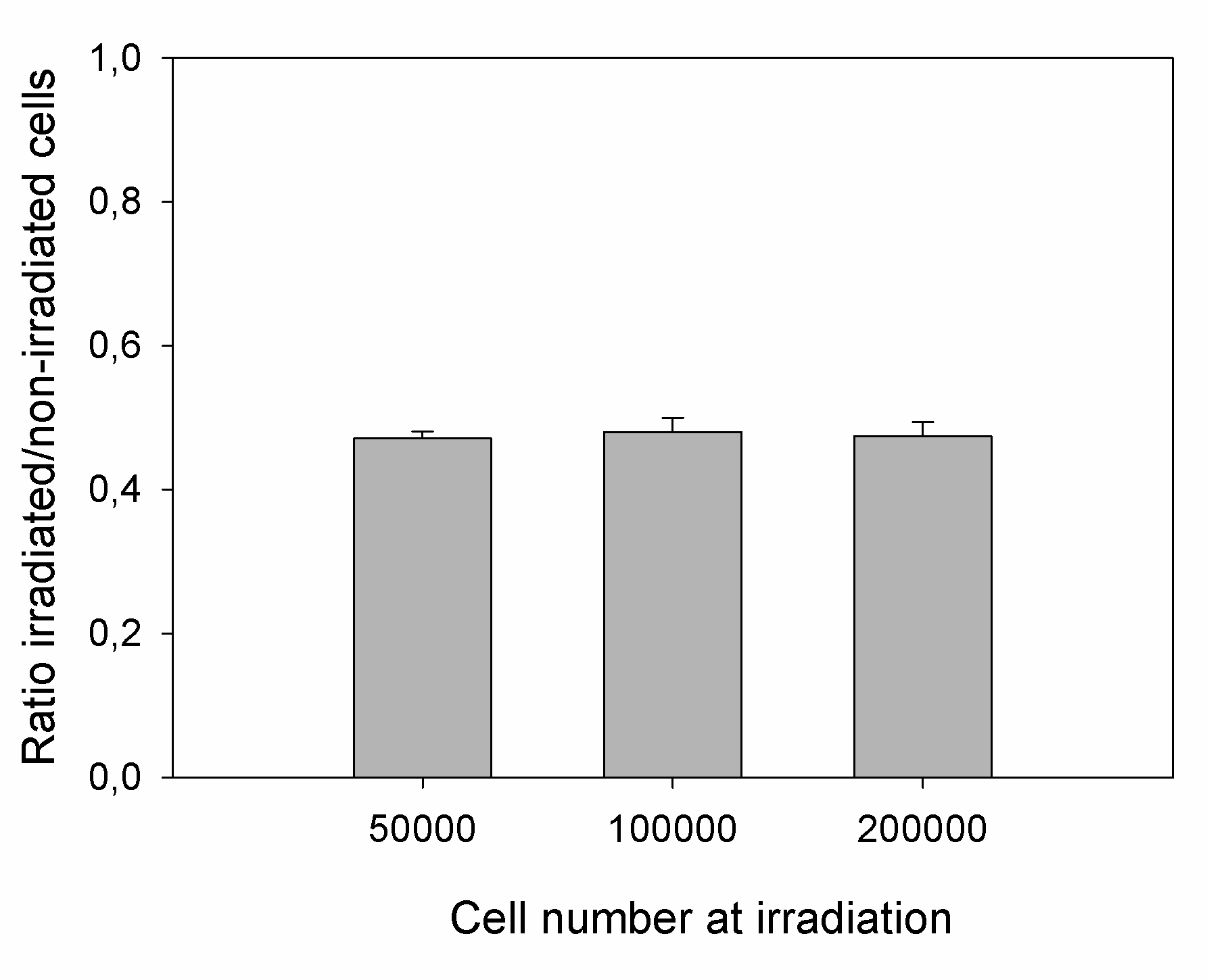

Supplement: Additional file 2 — Cell response to photon irradiation for various cell numbers. Cells were seeded in different confluences and incubated for 24 h under normoxia. Cell number was determined prior to irradiation. The ratio vital treated to vital untreated cells was determined 72 h after photon irradiation. Mean values and standard deviation. [file 1748-717X-6-35-S2.TIFF]
